# Person identification using the composition of elements in human hair

**DOI:** 10.25122/jml-2022-0100

**Published:** 2022-11

**Authors:** Saule Mussabekova, Xeniya Mkhitaryan

**Affiliations:** 1 Department of Pathology, Karaganda Medical University, Karaganda, Kazakhstan; 2 Department of Informatics and Biostatistics, Karaganda Medical University, Karaganda, Kazakhstan

**Keywords:** forensic medicine, hair samples, microelements, person identification, inductively coupled plasma atomic emission spectroscopy, IAEA – International Atomic Energy Agency, WHO – World Health Organization

## Abstract

If an individual cannot be identified, it is necessary to conduct a forensic medical examination. In this case, all possible group indexes are investigated. In this study, the content of elements in hair was investigated to identify individuals by territory, age, profession, or gender. The level of 14 micro- and macroelements (Cu, Zn, Co, Fe, Cr, Mn, Cd, As, Pb, Ni, P, Ca, K and Mg) was determined in hair samples of men and women from five age categories (21 to >60) using inductively coupled plasma atomic emission spectroscopy. The samples were analyzed taking into account the health condition, gender, place of residence, occupation, work experience, and age. A correlation between the content of elements in people's hair and their place of residence was observed. The difference in the content of elements in the hair of urban and rural residents was substantial and statistically significant (p<0.05). Moreover, there were significant differences related to age (p<0.0051) and gender (p<0.05). The current research detected significant differences in the content of the chemical elements in the hair of the groups tested, which can be used as personal identification indicators depending on occupation, work experience, and environmental factors.

## INTRODUCTION

We know the importance of chemical elements for people's physiology, but we have no clear understanding of their specific content [1]. For example, hair is an organ that effectively reflects the content of elements in a biogeochemical zone, which can serve as a diagnostic tool for mineral metabolism malfunction [2, 3].

Human hair, as a biological material, is appealing to forensic experts due to its ease of selection for study, long retention period, and informativeness [4]. In this regard, knowing the coefficients of the ratio of chemical elements in human hair is important, as well as information about the person's place of residence (permanent or temporary), occupation or profession, gender, and age. Analysis of the chemical composition of hair expands the possibilities of forensic medical identification of a person [5, 6]. Moreover, the content of elements in hair can be used for zoning areas and territories of industrial contamination and for determining the exact place of residence of a person [7, 8]. The study of regional features of the distribution and accumulation of essential elements (Cu, Zn, Co, Fe, Cr, Mn), toxic, conditionally toxic (Cd, As, Pb), and conditionally essential micro- (Ni) and macroelements (P, Ca, K, Mg) contained in the hair of men and women are of significant interest in solving the problems of diagnostics and identification of a person [1, 9].

Industry in the Republic of Kazakhstan has constantly been growing over the past ten years. The growth of coal mining amounted to 19.29%, oil and natural gas production grew by 18.59%, and metal ore mining increased by 26.43% [10, 11]. Furthermore, the Karaganda Region is one of the most polluted areas in Kazakhstan, with a pollution coefficient of 2.61 [12]. The total amount of contaminant emissions into the atmosphere has reached 593,000 tons of hazardous substances in Central Kazakhstan, representing 26.1% of the total air pollution in the country. People living in this region are affected by this situation [5, 11, 13]. A laboratory study of the content of chemical elements in the hair of people from this region can expand the possibilities of forensic medicine for identifying individuals by place of residence, age, gender, and professional features.

This study aimed to identify the qualitative and quantitative features of the mineral composition of human hair depending on gender, age, profession, and place of residence and to indicate the primary markers for the forensic identification of a person.

## MATERIAL AND METHODS

The content of elements in hair was studied in men and women from five age categories. For the last 5 years, all the people have lived on the territory of Central Kazakhstan in the cities of Karaganda (region 1), Temirtau (region 2), Balkhash (region 3), and Zhezkazgan (region 4). Hair samples of 217 corpses and 1238 living persons working in industrial and non-industrial sectors, living in urban and rural areas at different distances from the main sources of technogenic impact, were studied. The quantitative indicators obtained during the experiments are presented in Table 1.

**Table 1:** Place of residence and occupation of the examined persons.

Gender	Place of residence		Distance from the place of dwelling to production-industrial estates					Occupation (place of employment or profession)	
	City	Village	50 km	100 km	150 km	200 km	250 km	Production industry	Other spheres
**Male**	314	300	120	122	125	124	123	389	225
**Female**	324	300	125	124	124	126	125	236	388
**Total**	638	600	245	246	249	250	248	625	613

Hair samples weighing 0.1 g each were cut to the full length (directly at the root) in several places of the cervical part of the head and packed in paper envelopes, which were stored at room temperature in a dry place.

### Data collection

Sample collection was carried out following the rules adopted by the Ethical Committee of Karaganda Medical University (Kazakhstan). Personal data was obtained using a questionnaire based on the World Health Organization (WHO) recommendation. Personal data included gender, age, profession, region of permanent residence, lifestyle, diseases, and habits.

### Sample preparation

Before the analysis, the hair samples were rinsed with a nonionic detergent and deionized water. Acetone was used to remove surface contamination and degrease the hair (for 15 minutes). Then the samples were rinsed three times with deionized water. Following this, the samples were dried at room temperature for 30 minutes. Before the analysis, the prepared samples were kept in desiccators. Directly before the analysis, the hair samples were cut into small fragments. Samples were decomposed by microwave sample preparation, and 0.5 g of each sample was placed into a fluoroplastic sleeve containing 5 ml of nitric acid. The autoclave with the sample was placed in a microwave oven, and the sample was decomposed (mode: raising the temperature to 200°C for 5 min, holding for 5 min, then cooling to 45°C). The cooled autoclave was shaken to mix the contents. The dissolved sample was quantitatively transferred into a 15 ml test tube containing 1 ml deionized water and shaken constantly. Subsequently, each eluate was placed into a test tube, deionized water was added to reach a volume of 10 ml, then the tube was closed to mix the contents. Finally, 1 ml of an aliquot was taken out with an automatic measuring device, and 0.5% nitric acid was added to reach a volume of 10 ml. Data on the volume of the aliquot part and the dilution, name, and weight of the sample were entered into the spectrometer software.

The content of 14 chemical elements: Copper (Cu), Zinc (Zn), Carbon Monoxide (Co), Iron (Fe), Chromium (Cr), Manganese (Mn), Cadmium (Cd), Arsenic (As), Lead (Pb), Nickel (Ni), Phosphorus (P), Calcium (Ca), Potassium (K), and Magnesiun (Mg) was analyzed using inductively coupled plasma atomic emission spectroscopy using an “ULTIMA-2” spectrometer produced by Horiba Jobin Yvon following the methodological guidelines and requirements of the International Atomic Energy Agency (IAEA) [14]. The obtained data were compared with each other and with reference values obtained in the process of application of inductively coupled argon plasma [interquartile range (q25–q75)] atomic emission spectroscopy [15, 16]. The analysis results were entered into a database containing the medical and biological characteristics of the examined persons. Statistical analysis of the results was performed using Statistica 10.0 (StarSoft Inc., USA) and SPSS 20 program packages. All values were expressed as Me/q25–q75, and the results were expressed in micrograms per gram. Statistical comparison of the results was performed using the Kruskal-Wallis test (a non-parametric test for several independent samples) to evaluate the differences between the groups and the Mann-Whitney U test. Differences in values were considered statistically significant at a probability level of more than 95% (p<0.05) for two compared groups [17], p<0.0170 for three compared groups, p<0.0085 for four compared groups, and p<0.0051 for five compared groups [18].

## RESULTS

### Analysis of element concentration by vital status

There was no difference in the quantitative content of the 14 studied elements in hair samples obtained from living persons inhabiting a certain region of Central Kazakhstan and those from corpses of people who lived in the same region, regardless of gender and age. Furthermore, there were no significant differences in the hair samples of living and deceased persons (p>0.05). Thus, for further research, the results were merged into one group, and all the subsequent comparisons were made regardless of the vital status (alive or dead) of the examined persons.

### Analysis of element concentration by gender

The content of elements in the hair has its peculiarities depending on the gender of the owner, with the most distinct gender differences typical for macro elements. A pairwise comparison of the content of studied elements in male and female samples revealed that the level of the content, except for P, significantly differed between genders (p<0.05). There were no significant differences in the level of Phosphorus between men and women (p>0.05). However, the content levels of most elements were significantly different depending on gender. Further comparisons were conducted separately for men and women.

### Analysis of element concentration by place of residence

Comparative analysis of the levels of essential elements (Cu, Zn, Co, Fe, Cr, Mn), toxic and conditionally toxic (Cd, As, Pb), conditionally essential microelements (Ni), and macroelements (P, Ca, K, Mg) in the hair of residents of Central Kazakhstan, regardless of gender, showed that the levels of elements under consideration differed drastically depending on the place of residence. The median concentrations (Me/q25–q75) of the elements in the hair samples of men and women residing in cities and villages of Central Kazakhstan are as follows (Table 2).

**Table 2:** An elemental portrait of the population residing in urban and rural territories of Central Kazakhstan depending on gender, mkg/g.

Element	Values (20), (q25–q75)	Criteria	Men					Women				
			City n=314	Village n=300	Statistical significance of the differences			City n=324	Village n=300	Statistical significance of the differences		
					U	Z	p-level			U	Z	p-level
Cu	9–14	q25	130	13	0	21.9776	0.0000	81	10	0	23.1724	0.0000
		Me	140	14				100	10			
		q75	150	14				100	10			
Zn	145–206	q25	210	208	11486.5	17.2448	0.0000	210	198	0	21.9839	0.0000
		Me	218	210				212	200			
		q75	230	210				215	200			
Co	0.04–0.16	q25	0.18	0.17	888	22.6637	0.0000	0.17	0.16	1405.5	24.1965	0.0000
		Me	0.19	0.17				0.17	0.16			
		q75	0.19	0.17				0.17	0.16			
Fe	11–25	q25	40	33	0	21.9374	0.0000	35	26	0	22.5448	0.0000
		Me	43	35				35	28			
		q75	45	35				35.5	28			
Cr	0.2–0.96	q25	1.3	0.96	0	21.9144	0.0000	1.15	0.96	0	23.7534	0.0000
		Me	1.5	0.96				1.15	0.96			
		q75	1.6	1				1.15	0.96			
Mn	0.32–1.29	q25	1.6	1.4	149	22.9755	0.0000	1.5	1.3	150	23.9099	0.0000
		Me	1.8	1.4				1.5	1.3			
		q75	1.8	1.4				1.5	1.3			
Cd	0.02-0.13	q25	0.25	0.15	0	23.0039	0.0000	0.2	0.14	151	24.8004	0.0000
		Me	0.5	0.15				0.2	0.14			
		q75	0.5	0.15				0.2	0.14			
As	0.00–0.98	q25	1.3	0.96	3.5	22.6761	0.0000	1	0.95	0	23.0691	0.0000
		Me	1.3	0.99				1.1	0.95			
		q75	1.3	0.99				1.1	0.95			
Pb	0.38–1.67	q25	1.9	1.7	0	23.1581	0.0000	1.8	1.69	456.5	24.4986	0.0000
		Me	2	1.7				1.8	1.69			
		q75	2	1.7				1.8	1.69			
Ni	0.14–0.53	q25	0.7	0.5	0	23.0238	0.0000	0.5	0.15	44	22.7189	0.0000
		Me	0.9	0.5				0.6	0.15			
		q75	0.9	0.5				0.6	0.15			
P	128–181	q25	120	127	12	-22.641	0.0000	120	127	0	24.0955	0.0000
		Me	120	127				120	127			
		q75	123	127				120	127			
Ca	354–1619	q25	350	1200	0	-21.866	0.0000	1100	1550	0	22.7699	0.0000
		Me	350	1200				1100	1570			
		q75	500	1250				1100	1570			
K	29–433	q25	190	350	362.5	-21.5	0.0000	190	320	180	22.1239	0.0000
		Me	200	360				200	320			
		q75	250	370				200	320			
Mg	32–137	q25	50	100	215.5	-22.262	0.0000	100	130	0	23.9526	0.0000
		Me	70	100				100	130			
		q75	70	100				100	130			

The data are presented as Me/q25–q75, where Me – median; q25 – lower quartile, q75 – upper quartile.

**Figure 1:**
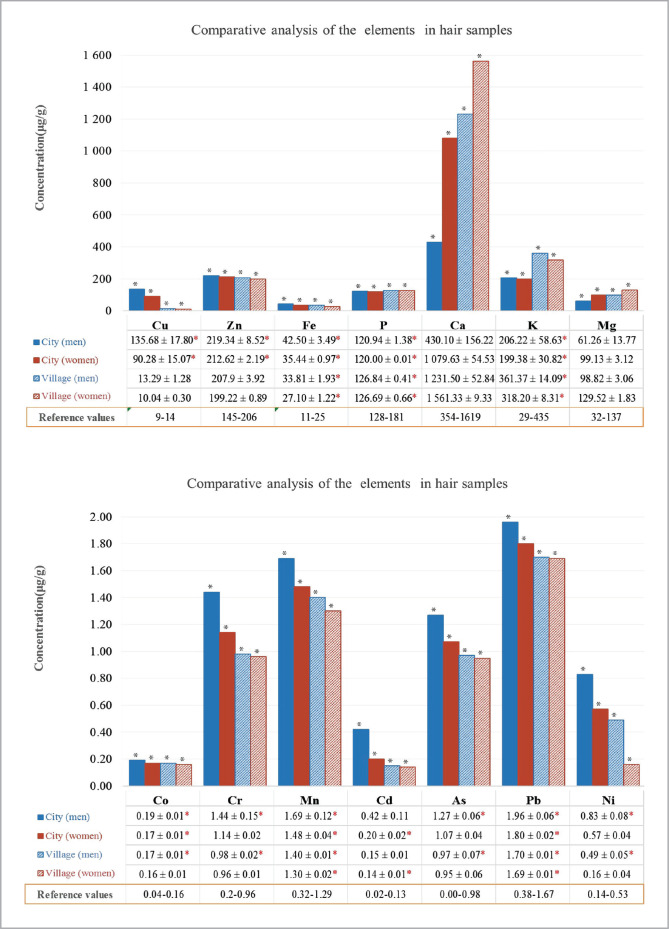
The analysis of the chemical elements in hair samples by gender and place of residence.

In men and women living in urban areas, the levels of microelements (Cu, Zn, Co, Fe, Cr, Mn, Cd, As, Pb, and Ni) in the hair were higher, whereas the levels of microelements (P, Ca, K and Mg) were lower than in the samples taken from the rural residents irrespective of gender (Figure 1). At the same time, the levels of all 14 studied elements in the hair samples of men and women living in cities were statistically different from the levels of the corresponding indicators in the hair samples from rural areas. When using non-parametric statistics (Mann-Whitney test), the calculated value of U did not exceed the critical value, which proves the presence of some differences between the groups (p<0.05). The results of the statistical calculations are presented in Table 2. The concentrations of Cu, Zn, Fe, Mn, Co, Cr, and Ni, and, especially, toxic Cd, As, and Pb in the hair samples of men residing both in cities and villages from Central Kazakhstan exceed the biologically acceptable levels (p<0.05), with each of these elements characterized by an individual range of fluctuations.

The levels of Cu and Zn content in the hair of rural women varied within the acceptable values (p<0.05), while in the hair of urban women, it exceeded the acceptable limits (p<0.05). Regardless of their place of residence, Ni and Cr content in the women's hair samples did not exceed the biologically acceptable levels (p<0.05).

It was determined that, regardless of gender, there was a deficiency of macroelements (p<0.05) in the hair samples of residents from Central Kazakhstan. Thus, the deficiency of Ca, K, and Mg were more pronounced in the hair samples of men and women living in cities than in those living in villages (p<0.05). However, it is worth mentioning that the content of P in the hair samples of urban residents did not differ from that in rural residents, regardless of their gender (p<0.05).

The most significant difference in the content of microelements from urban and rural samples was registered in the accumulation of toxic elements such as Cu and Fe. The concentrations of Fe and Cu in the hair of people living in cities exceed the corresponding average indicators in rural areas by 1.3 and 10 times, respectively. The hair samples of urban residents showed an increased concentration of Zn by 27%, Mn – by 31%, Co – by 16%, Ni – by 27%, and the concentration of As was 20% higher than in the hair samples of rural residents.

### Analysis of element concentration considering the remoteness of a person's place of residence from large industrial and production facilities

There were significant differences in the levels of chemical elements, regardless of gender, in the hair of the residents from more remote areas and the people living in relatively polluted areas of industrial and logistics complexes. The median level of the studied elements in the hair of all citizens residing 250 km from industrial production was closer to normal. However, as the distance between the place of residence and the location of industrial and logistics complexes shortened and the anthropogenic load increased, the quantitative characteristics of the studied toxic and conditionally toxic microelements showed significant growth. Their maximum concentrations were registered in people residing within a radius of 50 km from industrial production. At the same time, deficiencies of macroelements (P, Ca, K and Mg) were more pronounced in people residing closest to industrial production. The farther away from production, the less the deficiency. The Kruskal-Wallis test with test size (H=1196.2, variability – 4, N=1238, p=0.000 and H=1078.9, 4, N=1238, p=0.000) for Cu and Zn, respectively, proves the statistical significance of the influence of the remoteness of the place of residence from the main sources of pollution on the concentration of Cu and Zn in the hair of the local population. The pairwise comparison revealed significant differences between all the examined groups. Multiple pairwise comparisons and a median test indicated statistically significant differences in the decrease of Co, Fe, Cr, Mn, Cd, As, Pb, Ni, P, Ca, K, and Mg levels depending on the distance of the place of residence from industrial production in all studied groups (p<0.0051), except between 50 km and 100 km for Mg; 100 km and 150 km for Co and Pb; 150 km and 200 km for Fe, Mn and Ca; 200 km and 250 km for Cr, Cd, As, Pb, Ni, P, K and Mg (p>0.0051).

### Analysis of element concentration considering the professional activities of the residents

**Figure 2:**
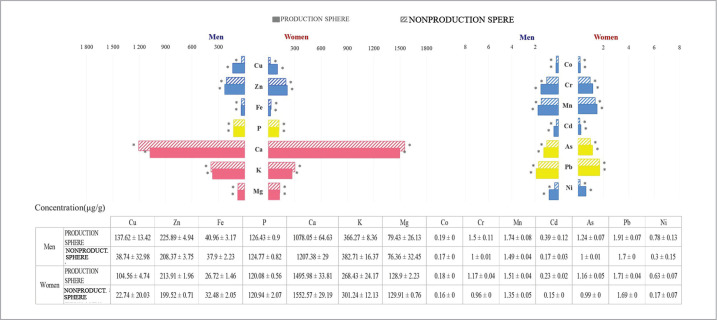
Comparative analysis of the levels of chemical elements in hair samples of men and women by the sphere of work.

The analysis of bioaccumulation and redistribution of microelements depending on the nature of work (occupation) revealed certain patterns (Figure 2). Thus, the level of the content of the studied microelements (Cu, Zn, Co, Fe, Cr, Mn, Cd, As, Pb, Ni) in hair samples, regardless of gender, was significantly higher in the samples of people employed in the manufacturing sector than those working in areas not related to the industrial complexes of the region (p<0.05). Deficiency of 3 out of 4 studied macroelements in the samples of men (P, Ca, K) and of all the studied elements (P, Ca, K and Mg) in the samples of women employed in production was more pronounced than in the samples of people employed in other areas (p<0.05). Data on the level distribution of the 14 studied elements depending on the nature of work are presented in Table 3. Although, it should be noted that the level of Mg in the hair of men, regardless of the nature of their work, remained stable (p>0.05). The calculated value of U in the Mann-Whitney test did not exceed the critical one, indicating the differences between groups (p<0.05).

**Table 3:** Content of chemical elements in the hair depending on the nature of work.

Element	Parameters	Men			Women		
		Production sector (n=389)	Non-production sphere (n=225)	Significance, p	Production sector (n=236)	Non-production sphere (n=552)	Significance, p
Cu	q25	130	14	<0.001	100	14	<0.001
	Me	140	14		103	14	
	q75	150	50		110	14	
Zn	q25	220	210	<0.001	214.5	199	<0.001
	Me	230	210		215	200	
	q75	230	210		215	200	
Co	q25	0.19	0.17	<0.001	0.18	0.16	<0.001
	Me	0.19	0.17		0.18	0.16	
	q75	0.19	0.17		0.18	0.16	
Fe	q25	37	35	<0.001	25	30	<0.001
	Me	40	39		27	33	
	q75	45	40		28	34	
Cr	q25	1.4	1	<0.001	1.1	0.96	<0.001
	Me	1.5	1		1.2	0.96	
	q75	1.6	1		1.2	0.96	
Mn	q25	1.7	1.5	<0.001	1.5	1.3	<0.001
	Me	1.8	1.5		1.5	1.3	
	q75	1.8	1.5		1.5	1.4	
Cd	q25	0.25	0.15	<0.001	0.2	0.14	<0.001
	Me	0.45	0.15		0.25	0.15	
	q75	0.5	0.2		0.25	0.15	
As	q25	1.2	0.99	<0.001	1.1	0.99	<0.001
	Me	1.2	1		1.2	0.99	
	q75	1.3	1		1.2	0.99	
Pb	q25	1.85	1.7	<0.001	1.7	1.69	<0.001
	Me	1.9	1.7		1.7	1.69	
	q75	2	1.7		1.7	1.69	
Ni	q25	0.6	0.15	<0.001	0.55	0.14	<0.001
	Me	0.8	0.25		0.65	0.14	
	q75	0.9	0.5		0.7	0.15	
P	q25	125	125	<0.001	120	120	<0.001
	Me	127	125		120	120	
	q75	127	125		120	120	
Ca	q25	1100	1200	<0.001	1500	1550	<0.001
	Me	1100	1200		1500	1560	
	q75	1100	1200		1500	1570	
K	q25	370	390	<0.001	250	300	<0.001
	Me	370	390		250	300	
	q75	370	390		300	300	
Mg	q25	60	25	0.793	130	130	<0.001
	Me	100	100		130	130	
	q75	100	100		130	130	

The level of Cu in the hair of men employed in the production industry was 10 times higher (in women, it was 7 times higher) (p<0.01), Ni – 3.2 and 4.6 times higher in the samples of men and women, respectively (p<0.05), Cd – 3 times higher in the samples of men and 1.7 times higher in the samples of women (p<0.05), Co, Cr, Mn, As, Pb – 1.1–1.3 times higher, depending on gender (p<0.001). However, no difference in the levels of the macroelements was found (p>0.05).

### Analysis of element concentration considering the duration of the professional activity and place of work

The examination of people working in the industrial production of the regions, regardless of their gender, was carried out in the following cities: Karaganda (region 1), Temirtau (region 2), Balkhash (region 3), Zhezkazgan (region 4). The examination revealed that the specificity of the toxic load was associated with the type of production. At the same time, it should be emphasized that in the hair samples of persons employed in the mining industry (region 1), a substantially increased content of Zn, Cr, and Pb was revealed. In the examined samples of people employed in metallurgical production (region 2), the levels of Fe, Cd, Co, Pb, and Ni were increased, and the workers of ore-dressing industries (regions 3 and 4) had higher levels of Cu and As, compared with people working in other regions or industries. In addition, an increase in work experience (length of service) in mines, metallurgical industrial complexes, or ore-dressing plants of Central Kazakhstan was generally accompanied by the accumulation of Zn, Co, Fe, Mn, As, Ni, as well as Cd and Pd in hair and a tendency to accumulate Cu (p<0.0051). It should be noted that the content of the macroelements was, at the same time, reduced (p<0.0051). Moreover, 20 years of work experience was associated with an increased content of Cu, Zn, Com Mn, Cd, As, Pb, and Ni in the hair of workers, and the experience of more than 40 years – of Cr (p<0.0051). The accumulation of these microelements and the deficiency of macroelements in the hair samples of workers of the abovementioned industries were directly related to the length of service in the industry.

### Analysis of element concentration considering the age

The concentration of some microelements in the hair samples of people working in production increased not only with the increase in work experience but also with age. It is curious that the increase or decrease in the studied elements occurred in various age periods and depended on gender. Data on the content levels of the macro- and microelements in 5 age groups are presented in Table 4. The level of Zn, Cr, Mn, As, Pb, N microelements, and K macroelement in the samples of men gradually progresses and reaches the maximum level by age 60. The median of the level of Cu and Cd reaches its peak by the age of 41 to 51, the level of Fe – by 51–60, the level of P – by 31–40, and then gradually decreases, while Co reaches its maximum by 31–40 and remains stable further on. It should be noted that the median levels of the content of macroelements such as Ca and Mg in the hair samples of men steadily decrease with age. The results of the Kruskal-Wallis test and the median test indicate statistically significant differences in the levels of Zn, Cr, Mn, Cd, Pb, Ni, and Ca between the age groups of 21–30 and 41–50, 51–60 and 60 and older; Cu and As are compared with the groups of 21–30 and 41–50 and >60; Co – with the groups of 21–30 and 31–40, 41–50 and >60; K – with the groups of 21–30 and 31–40, 41–50 and >60 in pairwise comparison with each other (p<0.0051). The content of P and Mg in the hair of men significantly differed between all groups (p<0.0051), and the level of Fe, on the contrary, did not differ in the studied hair samples of men within all 5 age groups (p>0.0051).

**Table 4:** Content of chemical elements in men and women depending on the age.

Element	Parameters	Men					Women				
		Age categories, years old					Age categories, years old				
		21–30 n=128	31–40 n=144	41–50 n=179	51–60 n=119	60 and older, n=153	21–30 n=133	31–40 n=144	41–50 n=147	51–60 n=152	60 and older, n=156
Cu	q25	20.5	23	35	13	31	10	21	12	21.5	30.5
	Me	68.5	68.5	107	36.5	40	100	66.5	100	69	39.5
	q75	122.5	124	110	148	110	120	122.5	139	120	107.5
Zn	q25	210	210	210	210	210	200	200	201	204.5	208
	Me	210	212	215	215	215	210	208.5	210	210	210
	q75	214	219	217	230	221	210	210	210	210	218.5
Co	q25	0.17	0.17	0.18	0.17	0.17	0.16	0.15	0.16	0.15	0.13
	Me	0.17	0.18	0.18	0.18	0.18	0.17	0.17	0.17	0.17	0.17
	q75	0.18	0.185	0.18	0.18	0.18	0.17	0.18	0.18	0.18	0.17
Fe	q25	30	32.5	34	30	30	25	25	25	25	25
	Me	35	35	35	37	35	30	30	30	30	30
	q75	37	38.5	37	45	37	39	36	35	32.5	32.5
Cr	q25	1	1.1	1.1	1.1	1.1	0.96	0.96	0.96	0.96	0.96
	Me	1.1	1.1	1.1	1.2	1.15	0.96	1	1	1	1
	q75	1.15	1.15	1.2	1.5	1.5	1	1.1	1.1	1.05	1.2
Mn	q25	1.4	1.4	1.4	1.5	1.4	1.3	1.3	1.3	1.3	1.3
	Me	1.4	1.4	1.5	1.6	1.6	1.3	1.35	1.4	1.3	1.3
	q75	1.475	1.55	1.6	1.7	1.6	1.4	1.45	1.5	1.4	1.4
Cd	q25	0.148	0.15	0.18	0.15	0.15	0.14	0.13	0.14	0.14	0.15
	Me	0.15	0.195	0.25	0.2	0.2	0.15	0.145	0.15	0.15	0.15
	q75	0.225	0.25	0.25	0.5	0.25	0.2	0.15	0.2	0.2	0.2
As	q25	0.45	0.79	0.98	0.99	0.99	0.1	0.54	0.3	0.3	0.88
	Me	0.99	0.98	1	0.99	1	0.98	0.98	0.99	0.98	0.98
	q75	1	1	1.3	1.1	1.1	1.2	1.05	1	1	1
Pb	q25	1.695	1.7	1.79	1.8	1.79	1.68	1.69	1.69	1.69	1.69
	Me	1.7	1.78	1.8	1.85	1.8	1.69	1.69	1.69	1.69	1.69
	q75	1.795	1.8	1.84	2	1.85	1.69	1.7	1.8	1.75	1.795
Ni	q25	0.18	0.4	0.5	0.5	0.5	0.14	0.14	0.16	0.18	0.18
	Me	0.39	0.525	0.53	0.7	0.65	0.25	0.355	0.18	0.315	0.56
	q75	0.575	0.6	0.6	0.9	0.7	0.6	0.58	0.7	0.635	0.57
P	q25	121	124	120	120	122	120	120	120	121	120
	Me	121	124	122	122	122	120	120	120	121	121
	q75	121	125	122	123	125	120	122	120	122	121
Ca	q25	1200	1100	700	500	310	1200	1470	1400	1200	1200
	Me	1200	1100	900	700	310	1200	1500	1500	1200	1300
	q75	1200	1470	1000	700	360	1470	1500	1550	1200	1400
K	q25	22	300	320	300	280	350	350	300	300	300
	Me	29	350	350	300	300	350	350	300	320	300
	q75	350	370	350	320	300	350	350	300	320	300
Mg	q25	100	50	50	60	50	100	120	120	120	120
	Me	100	100	70	70	50	100	120	125	120	120
	q75	100	100	80	70	60	125	125	130	127.5	120

The quantitative analysis of the 14 macro- and microelements levels in women's hair showed a completely different result. In this case, the median levels of Co, Fe, As, Pb, and P in the hair samples of women of the studied territory were higher than the reference values (p<0.05). Nevertheless, they remained stable throughout life. Cr, Cd, and Zn levels were relatively stable, with insignificant downward deviations during middle age (31–40). The median levels of Mn and Mg in women reach their highest values by the age of 41–50, and the level of Ca reaches its peak at the age of 31–40, remaining at this level up to 41–50, and only then it decreased, while the level of K was constantly falling with age. The unstable levels of Cu and Ni in women should be particularly mentioned, as they periodically increased and decreased throughout life in different age periods: for example, the concentration of Cu reached the maximum peak in the 21–30 and 40–50 age groups, and Ni in 31–40 and >60. There were significant differences between the age groups in various combinations only in the levels of Zn, Mn, Cd, As, Pb, Ni, P, Ca, K, and Mg (p<0.0051). It is worth emphasizing that there were no statistical differences in Cu, Co, Fe, and Cr among all age groups (p>0.0051).

## DISCUSSION

The process of human biomonitoring is often based on the analysis of human hair samples. In forensic identification, human hair samples provide information about the features and characteristics of a person. Hair was proven to have a number of advantages over other biological tissues. First, it accumulates chemical elements [1, 19], and it has a unique feature of long-term information storage regarding metabolic processes and their changes under the influence of environmental factors [2, 7].

An assessment of gender differences in the content of Cu, Zn, Co, Fe, Cr, Mn, Cd, As, Pb, Ni, P, Ca, K, and Mg in the hair of residents from Central Kazakhstan determined that the level of these microelements in the hair of men is higher compared to women. This result is consistent with the data of Tamburo et al. [9], Sal'nikova et al. [20], and Mussabekova et al. [5]. The levels of Cd and Pb in male and female hair do not always have statistically significant differences [21, 22]. In our study, the levels of Ca and Mg showed opposite trends, and the level of P in men's and women's hair differed only slightly. Similar information can also be found in the publications of Mikulewicz et al. [4] and Mohmand et al. [23]. There are several reasons for such a difference. Firstly, depending on gender, these elements have specific peculiarities in their hormonal metabolism [9]. Secondly, a more regular professional contact of men at work and in private life, differences in diet and physical activity, as well as the influence of an exogenous factor such as smoking and a lower physiological level of Ca antagonist elements Mg and Zn [23, 24].

From our point of view, more pronounced processes of microelements accumulation and deficiency of macroelements found in the hair of people living in urban areas of Central Kazakhstan, compared with similar indicators in the rural population, were associated with high technogenic pollution, significant transport emissions, and most importantly these were specific for each type of production content of emissions from industrial enterprises of the region. The increased content of some toxic microelements in human hair can be explained by a direct relationship between the content of certain elements in hair and their content in the environment [3, 25]. According to He et al., the total impact of a complex of contaminants on the population of an urbanized territory is 4.9 times higher than that in rural areas [26]. It is worth mentioning that the main industrial and logistics complexes make the largest contribution to the sum of individual factors affecting the inhabitants of Central Kazakhstan. Moreover, we believe that the differences found may be associated with differences in the diet and lifestyle of the urban and rural populations.

Modern researchers agree that the determining influence on the content of chemical elements in human hair is exerted by the place of residence of a person, particularly its remoteness from the main industrial facilities of the region. Most studies in the literature showed the normalization of the content of the elements in human hair as the impact of technogenic factors decreased [3, 24, 27], which is most likely a consequence of different anthropogenic loads. These data are consistent with the results obtained during our research. At a distance of 200–250 km from a hypothetical source of pollution, the concentration of chemical elements in the hair of the examined people varied within the normal range, sometimes with a shift towards the upper limit.

Our study demonstrated the increased concentrations of the studied microelements in persons employed in the manufacturing sector, compared with those employed in the non-productive sector, regardless of gender. The available literature also indicates that the concentration of metals in biological samples increases in people who have constant professional contact with them [8, 28, 29]. For many, the manufacturing sector is a potential source of exposure to Cu, Zn, Co, Fe, Mn, Ni, As, Cd, and Pb [30]. Professions such as miners, foundry workers, welders, mechanics etc, are at especially high risk of contact with Cd and Pb [20, 27]. Buononato et al. [26] and Cabral Pinto et al. [31] note an increase in the average concentrations of Pb in the hair samples of men employed in the metallurgical industry. Hu et al. [25], Pan and Li [32], and Semenova et al. [33] demonstrate a 10-fold excess of maximum allowable concentrations of Cu in the hair of ore-dressing workers. Men may come into contact with “harmful factors” much more frequently than women, contributing to the accumulation of microelements. Some authors suggest that the increased level of toxic microelements in the hair of men working in production may result from increased metabolism [28].

The level of Mg in the groups of men working in the industrial and non-industrial sectors did not differ significantly. Therefore, the difference in the content of Mg in the hair samples of the indicated group might not be caused by its displacement from the body due to the anthropogenic impact of environmental toxicants entering the body but by the high level of Mg in the body. This proves that the diet of men, regardless of the nature of their work, is more saturated with necessary macro- and microelements due to subjective preferences.

Along with reports on the impact of the workplace on the quantitative content of human hair, there is evidence that the levels of Cu, Mn, Cd, As, and Pb in the hair of men and women have significant differences depending on the length of service in the enterprise [24, 34]. Our research also revealed similar differences in the content of Cu, Zn, Co, Mn, Cd, As, Pb, and Ni in the hair of men and women working in production, depending on their work experience. This is due to prolonged contact with stressors at the workplace, a decrease in the body's ability to detoxify harmful substances, a failure of the body's compensatory mechanisms due to the biological aging of the body, and the development of chronic diseases [30].

There is evidence in the literature that the content of certain microelements (Zn, Cr, Cd, Mn, As, Ni, Cu, Pb) in hair increases with age [34, 35]. However, according to the results of other scientists, there is no clear correlation between age and accumulation of, for example, Cd and Pb [20]. Our experiments helped to determine that the process of chemical elements deposition in human hair with aging depends on gender. It should be noted that the level of microelements Zn, Cr, Mn, As, Pb, Ni, and macroelement K in men's hair reaches its maximum by the pre-retirement or retirement age and then, in most cases, decreases. The decrease in the level of microelements in the hair of men of this age is, in our opinion, explained by a decrease in vitality, a frequent tendency to a sedentary lifestyle, a change in diet, and bad habits due to the emergence or progression of chronic diseases. While the levels of Co, Fe, As, Pb and P in women exceed the reference values and are quite stable throughout their lives, the levels of Cr, Cd, Zn, Cu, and Ni fluctuate within a certain range depending on the periods of a woman's life associated with reproductive function.

The chemical composition of hair is influenced by various geochemical features of the place of residence, age, gender, professional activities, work experience, and health status. This increases its value as a source of additional information in solving many forensic problems regarding a deceased person.

## CONCLUSIONS

This research showed the different levels of microelements accumulation in the hair of residents from Central Kazakhstan. This allows us to identify a person (or a corpse) in the crimes under investigation. The analysis of hair samples of Kazakhs living in regions with different environmental conditions depends on the different local industries. We could determine the background levels of the concentrations of the chemical elements characteristic for each region. These can be used for comparison in forensic identification and for assessing the impact of environmental pollution on human health. Additional differences depended on the place of work and residence, gender and age, professional activities, and work experience. Subsequently, we could adjust and narrow the search area for missing persons. Identifying a person using the chemical composition of hair is a new stage in modern biometric forensics, which makes it possible to unambiguously verify a person after death by the unique composition of chemical elements in hair samples. At the same time, it is important to monitor the concentrations and proportions of elements that can differ significantly from person to person.
